# Experiences, Motivations, and Perceived Impact of Participation in a Facebook-Based Support Group for Caregivers of Children and Youth With Complex Care Needs: Qualitative Descriptive Study

**DOI:** 10.2196/33172

**Published:** 2022-07-06

**Authors:** Katherine Jennifer Kelly, Shelley Doucet, Alison Luke, Rima Azar, William Montelpare

**Affiliations:** 1 Health Centred Research Clinic Department of Applied Human Sciences University of Prince Edward Island Charlottetown, PE Canada; 2 Centre for Research in Integrated Care Department of Nursing and Health Sciences University of New Brunswick Saint John Saint John, NB Canada; 3 Psychobiology of Stress & Health Lab Department of Psychology Mount Alison University Sackville, NB Canada

**Keywords:** caregiver experiences, peer-to-peer support, social support, social media, children with complex care needs, Facebook group

## Abstract

**Background:**

Caregivers of children and youth with complex care needs (CCNs) often require considerable support to ensure the well-being of their families. Social media present an opportunity to better support caregivers through computer-mediated communication for social support. Peer-to-peer (P2P) support groups are a way in which caregivers are accessing needed support; however, the experiences of caregivers who use these groups and the perceived impact that participation has on caregivers of children and youth with CCNs are not known.

**Objective:**

This study aimed to explore the experiences of caregivers of children and youth with CCNs who use a Facebook-based P2P support group to communicate, understand their motivations to use the group, and investigate its perceived impact on knowledge of programs and services and sense of community belonging among caregivers.

**Methods:**

A qualitative descriptive design was used to explore the experiences and perceived impact of a Facebook-based (Meta Platforms) P2P support group for caregivers of children and youth with CCNs in New Brunswick, Canada. The group was launched on the web in October 2020, during the COVID-19 pandemic, and resulted in 108 caregivers joining the group. A web-based survey was distributed, and semistructured interviews were conducted in February 2021 with a subsample of members. Thematic analysis was used to identify and report patterns related to caregivers’ experiences and perceived impacts of participation.

**Results:**

A subsample of members in the Facebook group completed the web-based survey (39/108, 36.1%) and interviews (14/108, 12.9%). A total of 5 themes emerged from the interviews: safe space, informational support and direction, web-based connection with peers, impact on knowledge of programs and services, and degree of community belonging. Participants reported joining the group to obtain geography-specific information support and connect with peers. Many participants reported an improvement in their knowledge of programs and services and felt connected to the community; however, the short observation period and diversity among the caregiver population were cited as barriers to community belonging.

**Conclusions:**

Social media present an important opportunity to facilitate the exchange of support between patients and caregivers in an accessible and curated environment. Findings from this study suggest that involvement in web-based, geography-specific P2P support groups can influence perceived knowledge of services and resources and sense of community belonging among caregivers of children and youth with CCNs. Furthermore, this study provides insight into the experiences and motivations of caregivers of children and youth with CCNs who participate in a private social media environment.

## Introduction

### Overview

Despite representing only 15% to 18% of the childhood population, children and youth with complex care needs (CCNs; aged 0-25 years) account for a substantial portion of health care costs and resource use in Canada [1]. Although pressure on the resources needed to treat these conditions is challenging the sustainability and effectiveness of Canadian health care systems, it also affects the well-being of children and youth with CCNs and their caregivers. Caregivers of children and youth with CCNs (eg, parents, guardians, and extended family members) face numerous challenges and barriers [2]. Obstacles faced by caregivers of children and youth with CCNs include the following: managing care from multiple providers and services, lack of information and access to resources, and emotional challenges [3,4]. These challenges have been exacerbated by the COVID-19 pandemic, leading to increased caregiver stress and loneliness [5]. For example, caregivers of children and youth with CCNs have experienced reduced access to and delays in health and social care, because of the pandemic [6], and increased stress owing to their child’s immunocompromised status [7]. Web-based peer-to-peer (P2P) support groups through social media are a way in which caregivers of children and youth with CCNs are accessing needed support [8]. However, the experiences of caregivers who use these groups and the perceived impact that participation has on caregivers of children and youth with CCNs are not known. This study aimed to explore the experiences of caregivers of children and youth with CCNs who use a geography-specific Facebook-based (Meta Platforms) P2P support group and investigate its perceived impact on knowledge of programs and services and sense of community belonging.

### Background

Caregivers of children and youth with CCNs often require considerable support to ensure the well-being of their families. Additional pressures on these caregivers can result in significant stress and isolation, particularly when attempting to navigate the health care system [3]. Social media websites and applications offer an opportunity to better support caregivers through computer-mediated communication for social support [9]; specifically, social media–based P2P support. Web-based support groups provide an environment for the exchange of informational, emotional, and instrumental support [9-11]; however, caregivers of children and youth with CCNs report primarily using these groups as a source of informational support [12]. Despite the prevalence of social media platforms available to users, Facebook, YouTube, and Twitter remain among the most popular websites for health-related P2P support [13].

Web-based P2P support offers an accessible and inexpensive source of informational knowledge and emotional support for caregivers [14], such as parents of children and youth with CCNs [15]. Caregivers of children and youth with CCNs who participate in web-based P2P support can acquire specific advice for their circumstances [9] and often consider the experience to be more relevant to their needs than the information provided by their professional care providers [16]. In some cases, the information exchanged within these groups goes beyond the knowledge of care providers, particularly for conditions that may be rare, not well understood, or beyond the scope of physical health care (eg, how to dress an infant with a feed tube) [17]. Web-based P2P support groups have been reported to supplement information received from a care provider [18-20] and help patients prepare for medical appointments [21]. In a 2014 survey involving parents of children with neurofibromatosis type 1, parents indicated that they were very likely to use internet P2P groups to seek research studies (87%), talk to parents with similar diagnoses (67%), and obtain answers to questions (50%) [22].

Although these communities are not meant to replace professional health care [23], they provide several important benefits to caregivers and their families. P2P support groups can promote access to information and create a sense of community belonging in patients and caregivers [24,25]. Web-based support can increase feelings of control, reduce isolation, and lower depression and anxiety in caregivers of children and youth with CCNs [26]. Health-related communication is often associated with risks including the privacy and reliability of information [27,28] and members’ ability to appraise relevant information [29]. However, observations of P2P support groups suggest that misinformation is often self-corrected over time by members who validate or correct the posted information [30]. Moderators have also been identified to play an important role in decreasing the spread of misinformation in groups [31].

One of the strongest motivations to engage in health-related P2P support is the desire to connect with individuals in similar situations [27]. Dumaij and Tijssen [32] reported four characteristics that play a role in an individual’s decision to use a particular website to connect with peers: (1) whether it is a closed-access website (ie, private), (2) nature of topics discussed, (3) ease of use, and (4) type of users and structure of discussions. Engagement with these groups can differ depending on various factors, including their target population. For example, parents of children with CCNs report using geography-specific groups for locally based informational or navigational support and condition-specific groups (eg, autism) for support specific to their child’s symptoms or diagnosis [8].

Belonging to a social group that is valued by contributing members can lead to a shared social identity [33]. This sense of social connectedness is an important consideration in P2P support groups that target caregivers of children and youth with CCNs. A poor sense of belonging has been associated with low caregiver well-being, which can affect the health outcomes of their child or children [34]. Lack of social belonging, or social isolation, can be defined as “a state in which the individual lacks a sense of belonging socially, lacks engagement with others, and has a minimal number of social contacts” [35,36]. Web-based platforms used for P2P support can promote a sense of social inclusion and belonging among informal caregivers [35], such as older adults [37,38]. The impact of these groups on caregivers of children and youth with CCNs specifically has not been previously explored; however, face-to-face parent support groups have been shown to increase the sense of community belonging among these caregivers [39].

Health literacy, broadly known as the ability to read and understand health information [40], is associated with knowledge of health-related services and has been identified as a barrier to navigating the health care system [41]. Low health literacy presents additional barriers when interacting with professional care providers, who often assume a higher level of understanding than an individual might possess [42]; this can be problematic for caregivers of children and youth with CCNs who often manage the care of their child [43]. Web-based P2P support offers an opportunity for individuals to engage with health information in a variety of ways, which can promote access to information [24] and improve knowledge of health-related resources [44,45]. Associations between web-based P2P support and health-related knowledge have been observed in breastfeeding mothers of preterm infants [20] and caregivers of persons with type II diabetes [46]; however, it has not been previously explored among caregivers of children and youth with CCNs. Specifically, the extent to which participation in web-based P2P support, through social media, affects health-related knowledge within this population is unclear.

### Objectives and Research Questions

The primary objective of this study was to explore the experiences, motivations, and perceived impact of involvement in a geography-specific P2P support group on Facebook and the motivations to use these groups among caregivers of children and youth with CCNs. More specifically, this study aimed to investigate the impact of participation in a group based in New Brunswick, Canada, which targets caregivers of children and youth with CCNs in New Brunswick, on perceived knowledge of resources and programs and sense of community belonging. The following research questions formed the basis for this study:

1. What are the experiences of caregivers of children and youth with CCNs who use the Facebook group to communicate with other caregivers?

2. Why do caregivers of children and youth with CCNs use the Facebook-based P2P support group?

3. In what ways does participating in the Facebook group affect the perceived knowledge of services or resources among caregivers of children and youth with CCNs in New Brunswick?

4. In what ways does participating in the Facebook group affect the perceived sense of community belonging among caregivers of children and youth with CCNs in New Brunswick?

## Methods

### Design and Sample

A qualitative descriptive design was used to explore the experiences and perceived impact of a Facebook-based P2P support group for caregivers of children and youth with CCNs in New Brunswick. Qualitative description is a pragmatic qualitative approach that facilitates obtaining simple, straightforward answers to questions in applied health research [47], while offering a comprehensive summary of an event or experience in everyday language [48].

A Facebook group was launched in October 2020; the details of this group have been described in 2 other publications [12,49]. Briefly, a group was created on Facebook, specific to caregivers of children and youth with CCNs who live in New Brunswick, Canada. Prospective members were screened at the time they provided consent for the study. Content in the group (including posts and the membership list) is closed to current members; however, the title, description, and profiles of moderators are visible to the public. The group was designed in collaboration with the NaviCare/SoinsNavi’s Family and Patient Advisory Council (PFAC), which provided insight into the following variables: language, group description and title, moderators, recruitment strategy, research observation, and evaluation. The PFAC consists of 6 parents of children and youth with CCNs and 1 young adult who experienced CCNs as a child; this council advised the research team at each stage of the research process to ensure its relevance to the target population. Group content is available in English and French and is closed to members (ie, private). The group was moderated by a member of the PFAC and the NaviCare/SoinsNavi patient navigator; the navigator provided support in both English and French. A description of the use of the group by caregivers and the factors that influenced group activity (eg, posts and interactions) have been published elsewhere [12].

Caregivers of children and youth with CCNs were recruited through advertisements on other relevant Facebook groups (eg, New Brunswick–specific groups for parents), media releases to relevant community organizations (eg, NaviCare/SoinsNavi), and word of mouth. The group attracted a total of 108 caregivers over the 6 months of the study period and has been primarily used by members to find answers to inquiries related to their child’s care and for the exchange of informational support, such as navigational support [12].

### Data Collection and Analysis

A web-based survey was distributed to members of the Facebook group in February 2021, which consisted of 19 questions related to their experience in the group. Items for the survey were developed for the purpose of this study and were pilot-tested among the PFAC and research team members for comprehension. The survey consisted of items in four categories: (1) sociodemographic information (including information about their child or youth with CCNs, such as age, condition or diagnoses, etc), (2) social media use (including how often they visit Facebook, membership with other health-related Facebook P2P support groups, etc), (3) use of the Facebook support group (including length of membership, visibility of content from the group on participants’ time line, frequency of interactions in the group, motivation for joining the group, etc), and (4) perceived impact of group membership on knowledge of services and resources and sense of community belonging (eg, “Have you learned about any services or resources for children or youth with health care needs in New Brunswick as a result of your membership in [the Facebook group]?”). The lead author (KJK) conducted the interviews using the Zoom videoconferencing software in February 2021 and March 2021; interviews lasted between 25 and 40 minutes. A pilot interview was conducted with a patient navigator from NaviCare/SoinsNavi in February 2021. Interviews were recorded using Zoom and transcribed verbatim manually by the lead author.

Members of the P2P support group were invited to participate in the survey through a direct link that was pinned to the top of the group. The bilingual survey was developed using Qualtrics Experience Management (Qualtrics International Inc). Semistructured interviews were conducted with a subsample of members in the group in February 2021 and March 2021 using Zoom videoconferencing software. Similarly, interview participants were recruited from existing members of the Facebook group and from members who indicated in the survey that they would be interested in participating in a follow-up interview. Interview participants were required to have been a member of the group for a minimum of 3 months; this was confirmed with participants by a direct message before scheduling an interview. All interview participants received a CAD $25 (US $19.32) Amazon gift card as compensation. Participants who completed the survey were entered into a draw to receive a $50 (US $38.63) Amazon gift card.

Open-ended survey questions and interview transcripts were analyzed using thematic analysis [50], as a means of identifying, analyzing, and reporting patterns across the data set and organizing and describing the data in rich detail [51]. Specifically, the lead author read through the transcripts and assigned initial codes to the content. Codes and associated quotes were collected in Microsoft Excel to produce a summary table [51] and grouped into broad themes using an iterative process to ensure that the original contexts of the quotes were preserved. Microsoft Excel was used to analyze both the quantitative and qualitative data.

### Ethics Approval

This study was approved by the University of New Brunswick’s Research Ethics Board (040-2019).

## Results

### Web-Based Survey: Demographic Information

A total of 36.1% (39/108) of the individuals who were members of the Facebook group completed the web-based survey. Most survey participants were women (29/39, 74%), and the remaining participants (10/39, 26%) chose not to answer. All the participants (39/39, 100%) were aged >25 years, with 41% (16/39) reporting their age between 25 and 44 years. Only 3% (1/39) of the participants was aged >55 years.

Most survey participants (21/39, 54%) reported caring for 1 child or youth with CCNs, 23% (9/39) of the participants reported caring for 2 children, and the remaining 23% (9/39) of the participants did not provide a response. Most participants reported caring for children aged 6 to 12 years (13/39, 33%), followed by children aged 4 to 5 years (11/39, 28%). Participants reported caring for 4 young children aged between 2 and 3 years and 4 youths aged between 13 and 18 years (Table 1).

**Table 1:** Age (in years) of children or youth under the care of survey participants (N=33).

Demographic	Children or youth, n (%)
0-1	1 (3)
2-3	4 (12)
4-5	11 (33)
6-12	13 (39)
13-18	4 (12)

Participants were able to select multiple responses if they were caring for >1 child or youth.

Conditions identified by caregivers were grouped according to 6 categories: mental health conditions (8/39, 21%), developmental conditions (16/39, 41%), neurological and genetic conditions (9/39, 23%), movement and motor conditions (8/39, 21%), cancer (1/39, 3%), and undiagnosed CCNs (7/39, 18%). The most common type of mental health condition included anxiety (3/39, 8%) and attention-deficit/hyperactivity disorder (2/39, 5%). Autism (9/39, 23%) was the most commonly reported developmental condition, followed by global developmental delay (4/39, 10%). Neurological and genetic conditions consisted of 9 different very rare conditions; these are not reported to protect the anonymity of participants in the study. Cerebral palsy (7/39, 18%) was the most common movement condition. The total number of conditions reported exceeded the number of survey participants (n=39), as approximately one-third of participants (12/39, 31%) reported caring for a child with multiple diagnosed conditions.

### Web-Based Survey: Motivation to Participate and Perceived Impact of Participation

Most survey participants reported becoming aware of the group through a friend or acquaintance (11/39, 28%) or through NaviCare/SoinsNavi (7/39, 18%). A total of 10% (4/39) of the participants reported learning about the group through another support group on the platform. When asked about their motivations for joining the Facebook group, the survey participants reported the topic to be relevant to their needs (23/39, 59%), the need for information or support (16/39, 41%), and the desire to make connections with others (13/39, 33%), among other reasons (Table 2). *Other* reasons included the foresight to use the group as a resource for future support needs.

**Table 2:** Indicated motivation or motivations for joining the Facebook group (n=76).^a^

Reason for joining the group	Participants, n (%)
The topic is relevant to me	23 (30)
I was or am in need of information or support	16 (21)
I was or am looking to make connections with others	13 (17)
The content appeared to be trustworthy	10 (13)
It is an active group	10 (13)
A mutual friend invited me	3 (4)
I heard about the group offline	1 (1)

^a^The total number of motivating factors (n=76) exceeds total survey participants (n=34) as participants were able to choose multiple responses.

Approximately one-third of respondents (14/39, 36%) indicated that they had learned about new services or resources relevant to their child’s or children’s care from participation in the Facebook group. Another 31% (12/39) of the participants indicated that they did not learn anything new. Totally, 10% (4/39) of the participants responded that they did not know whether they had learned anything new. When asked about the impact of the group on caregivers’ role in caring for their child or children using an open-ended question, 5% (2/39) of the participants stated that the group improved their sense of community belonging. None of the participants in the surveys or interviews (0/39, 0%) reported that the group negatively affected their knowledge of services or resources or sense of community belonging.

### Thematic Analysis of Interviews

#### Description of Themes

A total of 12.9% (14/108) of the participants who were members of the Facebook group completed the interviews; all interview participants also completed the web-based survey. A total of five themes emerged from the interviews that related to caregivers’ experiences in the Facebook group and the perceived impact that being a member had on their knowledge of services and resources and sense of community belonging. The themes were as follows: (1) safe space, (2) informational support and direction, (3) virtually connect with peers, (4) impact on knowledge of programs and services, and (5) degree of community belonging. These themes are described in further detail in the following section.

#### Theme 1: Safe Space

Participants described their experience in the Facebook group as a positive environment for the exchange of P2P support. Many participants characterized the group as a safe space that was inclusive of all caregivers, regardless of conditions or diagnoses:

I feel like this space is inclusive to everyone at different levels, in their diagnosis and in their journey.

Participant 10; March 4, 2021

Compared with other Facebook support groups, this group was considered to be safe by some members owing to its specificity to caregivers of children and youth with CCNs and the culture in New Brunswick:

I find sometimes with like, for instance, my [condition specific] group and things like that it's people all over the world. So, you know, I understand that sometimes things aren’t translated the same? [laughs] Or the intentions are not the same, or sometimes, you know, people can comment on something and it meant to be good, but you read it, you're like, ‘oh, okay, that was saucy, or that was like,’ you know what I mean? But I find this Facebook group, I don't see any of that, we're all kind of, at the same, you know what I mean? Like, ...it's in New Brunswick. It's here, I could bump into you at Costco or...I could meet them for coffee somewhere. Their kids could meet ours you know what I mean?

Participant 14; March 18, 2021

When initially joining the group, some members reported feeling inadequate or doubtful about their place within the group, which they referred to as the imposter syndrome. However, these participants explained that this quickly dissipated after spending time in the group:

[My friend] messaged me [that] this group actually just started, you should join it [laughs]. So, I did and then I immediately got, I think it’s called, is it imposter syndrome or something? Cause I just, like to me cancer is no big deal anymore and all these children that are, like, to me, are ‘real special needs,’ which I know is not, like, the right way to look at it, but it’s just the way that I, the brain works. So I definitely feel, not intimidated, not the right word, but I just felt like, oh like, we don’t belong in this group, right away. But I’m over that now [laughs].

Participant 07; February 23, 2022

#### Theme 2: Informational Support and Direction

Many participants described significant gaps in their support needs, particularly related to informational needs and navigational support regarding relevant programs, services, and resources. In some cases, participants reported being provided with an overwhelming amount of information upon recognition of a condition or diagnosis and left to determine the next course of action:

I think the thing is that once you get your child's diagnosis, for me, I felt like I was given pamphlets, I was given appointments, like you're being pulled, like your life just was just turned upside down. And you're given all this information and sometimes you just don't know what to do with it. It slips through the cracks, you're grieving, you're processing, you're trying to figure out all of a sudden, you know, you thought your life was going one way with a child and all of a sudden, it's like, whoa, now it's brand new...So you're trying to figure it out. And it took a lot of my husband and I having to figure it out calling and asking questions and making sure that we weren't missing something, and it's exhausting...We all have children with disabilities that we are trying to get the best care for and offer them the best quality of life. And I feel like that the [NB] group is set up to support [us] in that.

Participant 14; March 18, 2021

Participants described the mental load associated with being a caregiver of a child or youth with CCNs and explained that the Facebook group has been an important informational resource to help ease some of the pressure:

So going to that Facebook page and then there’s people coming to it with questions and right away someone says ‘well I did this’ ‘I did that’ and I think, wow, that’s, that’s, you know. Those are the hours and hours that I spent looking for information where now I can go and look and see someone’s experience. That narrows my search into ‘I’ll try this first, if it works, great. If it doesn’t, I can at least, you know.’ Where I didn’t even know where to start [laughs].

Participant 11; March 4, 2021

Specifically, the group was viewed as an important source of informational support, one that could provide a starting place and direction in the overwhelming amount of information provided to caregivers when their child or children experience a new diagnosis or crisis:

Just getting that advice from others parents is huge and it kind of helps you direct yourself. When it’s very overwhelming, that kind of gives your brain a place to like, settle on, and then “OK how do we approach this” and then it usually spirals, you can get a lot more information.

Participant 06; February 19, 2021

Having the Facebook group is helpful, where it's like, ‘Oh, I didn't realize that.’ Maybe we were given the information at first, but we forgot about it, or misplaced it, or...you didn't think that that was applicable to you at that time and you were just so heavy in the grief.

Participant 14; March 18, 2021

Many members described a need for informational support owing to an expressed lack of control that is associated with caring for a child or youth with CCNs. More specifically, seeking informational support was described to elicit a sense of empowerment:

There’s a lot of lack of control when you have a kid with special needs. I’m a control freak, [my husband] will say that. So I feel some sense of control and some power in her diagnosis if I have more knowledge of it. So if I know this is what we need to do or this treatment might help or whatever, whatever, it makes you feel like you have a little power in a very powerless situation. [The group] is a nice avenue to have if I have questions.

Participant 04; February 18, 2021

Participants described feeling reassured by their membership in the group, knowing that it was a place that they could turn to for support if and when needed:

I find that even just having the Facebook group, just having it there is helpful. Just knowing that you can comment or post if you need to post. Like, just having it there.

Participant 02; February 17, 2021

#### Theme 3: Virtually Connect With Peers

Participants described a desire for a group specific to caregivers of children and youth with CCNs in New Brunswick before the implementation of the study group. A participant explained having attempted to start a support group in the past, which was not successful:

I have been searching for this type of support for the last 6 years, even to the point of trying to start my own group, which was a super flop. I very much appreciate confidentiality of medical situations, but I think that was the biggest barrier. The therapists and doctors that everyone saw were unable to connect people together and there is no place to put up a poster or advertise really just to look for other real people, not just professionals who help, who are going through similar circumstances. I love the fact that it is a small, provincial group. I never would have guessed there were so many people here! I really felt like we were the only ones for a long time. The only people who even knew someone who had a complex need that is. And that is real lonely.

Participant 03; February 17, 2021

Many participants were motivated to join the Facebook support group to engage in communication with individuals who were experiencing similar situations and understood their challenges:

You know, something could happen with a child that morning and they get through it with the doctor, blah, blah, blah, and then they want to talk about it...And you can’t talk to anyone but your own family members, and friends, but...they haven’t lived your life. I think with this group you’re able to say, I need some help. And people are doing that, so that’s good.

Participant 11; March 4, 2021

More specifically, the solidarity associated with membership in a group of peers facing similar challenges in the same province was identified as an important reason why some participants used the group:

Which is helpful, because you have your support of your family and friends and that’s always valuable, but the support that you get from people who are going through a similar journey is just a different, you just feel heard, and you feel valued, and you feel understood, even if it’s online, it’s very, very helpful. I don’t think anything could replace that, especially when you have children that have any type of rare syndrome, you might not meet anyone that has that syndrome. So it’s been a benefit...just having the [NB Facebook] group community, a huge support.

Participant 06; February 19, 2021

When my daughter first got her chair, I wish we could have talked with other people too. I think there is a lot to gain from talking with people who are living the experience and not just professionals who support you. Not just about the facts of wheelchair life, but just knowing that there are other people going through the same challenges and success as you and connecting with them.

Participant 03; February 17, 2021

Some participants pointed to the web-based aspect of the support group as an important factor for their use. The availability and accessibility of the group were perceived as particularly important facets by caregivers, many of whom felt overwhelmed by the daily pressures associated with raising a child or youth with CCNs:

As a caregiver, it's completely different. You're burnt out, you're tired at the end of the day, you don't want to go to a support group. You...just want to sit if you can [laughs]. We're talking parents that are...doing heavy lifting still with their four or five teenage kids, you're talking parents that are doing diaper changes...anything that's in a routine for kids is more complex for us.

Participant 14; March 18, 2021

#### Theme 4: Impact on Knowledge of Programs and Services

Participants described engaging in web-based research of resources and information, which often occurred during the early stages of a condition or diagnosis. Participants reported feeling that they had a good understanding of the available programs and resources for their child or children. However, most participants speculated that there may be additional resources and programs beyond their knowledge, owing, in part, to their difficulties in navigating among services:

I feel like I know about a lot of them, but I also don’t know about a lot of them. Like, even through the Facebook group and...through other doctors or people, I’m still learning about things. Or maybe something that’s available in another province that’s just starting in New Brunswick or should be available in New Brunswick too and like, things like that.

Participant 03; February 17, 2021

When asked about their perceived impact of membership in the group on their knowledge of programs and resources, many participants reported feeling that it had improved their awareness of the available support:

It's only been five months [in the group], but in our case we've already searched for resources. We managed to find some but I imagine that parents who have just learned that their child is sick with disabilities, it will help them.

Participant 1; February 17, 2021; French translation

Participants described learning about programs and services by reading posts made by other members and directly making inquiries to the group. Some participants reported learning about programs and services that may be relevant to their child’s needs, but were located in other parts of the province. However, learning about programs or services that may not be applicable to their specific geographic location was described as providing an opportunity to ask if anyone knew about similar services in their region:

I definitely learned about more. Not all of them in my area, but just knowing that other parts of the province makes me feel like, I could still maybe ask of some. Um, yeah, I’ve definitely been more aware of different programs.

Participant 03; February 17, 2021

Some participants reported no increase in their knowledge of available programs and services through participation in the group, but instead reported perceiving the group as a place where they could go to if they had specific questions related to programs or services:

I haven’t hit a groove yet that this has improved it, or I’ve felt supported, but I also wouldn’t say that I’m not going to follow this page anymore cause I’m not interested. So I would say that I’m middle of the road on that.

Participant 08; February 23, 2021

In some cases, these individuals felt that they did not know what support they needed and lacked the language to ask for informational support about the available services and programs. In other words, participants described feeling uncertain about the types of services or programs that may exist or be beneficial to them in the care of their child or children: 

We haven’t found any resources. And to say that, I couldn’t even tell you an example of what we’re looking for because I don’t, I’m a first-time cancer mom, so I actually don’t know what resources I am seeking out.

Participant 07; February 23, 2021

#### Theme 5: Degree of Community Belonging

The extent to which caregivers of children and youth with CCNs felt that they belonged to a community within the P2P support group varied. Despite the short length of time since the inception of the Facebook group, most participants reported feeling a sense of community belonging within the group:

It’s definitely just helped me to see that there’s a lot of families in New Brunswick, a lot more than you think...are in the same-ish boat that you are in. I thrive off of community now that we’re in this situation. I just, I love to just talk to other parents who are feeling the same thing, it reassures me, it makes me think that I’m not alone in this crazy ordeal. Um, so to me, I just like to be a part of this group.

Participant 07; February 23, 2021

Some members attributed this reported sense of community belonging to the group’s membership exclusivity. More specifically, the group was private and only permitted caregivers of children and youth with CCNs who reside in New Brunswick:

It's made me feel more connected to our province, knowing that there are other parents out there going through, you know, similar experiences. ‘Cause a lot of the networks that I’m a part of are either like, Canada wide, or you know, different countries, so it’s nice to be in a group that’s just New Brunswickers.

Participant 10; March 4, 2021

More specifically, despite differences in the ages and conditions of their children, the shared experiences among caregivers were reported to facilitate a sense of community belonging within the group:

Even, even if you don’t post a lot, ...it just feels like you’re a part of...something similar, even if it’s not even the same thing. It’s similar enough that people understand the medical stays, the hospital stays, they understand the day to day, how much extra you do in a day. So, I think that, just that, initially creates an initial sense of community support.

Participant 06; February 19, 2021

A total of 29% (4/14) of the participants reported that they did not feel a sense of community belonging within the group. These individuals attributed this lack of community belonging to the short time since the implementation of the group. Some members described the same reason for experiencing few social ties with other members within the group. However, these individuals reported that they may benefit from a sense of community belonging over time:

I think that the relationship is still very new and very fresh...I think that it’s something that will, that has benefitted me and will continue to benefit me and my family, so yeah.

Participant 05; February 19, 2021

A participant, who reported feeling disconnected from the web-based group, explained that they did not identify with other members, many of whom are caring for young children:

I’d be more interested if something came across my Facebook page from somebody who might be 55 with a 30-year-old and what they’re doing for care and support...I haven’t seen a lot of that.

Participant 08; February 23, 2021

## Discussion

### Principal Findings

Consistent with previous studies, most participants reported using the group as an important source of informational support in the care of their child or children [52]. Findings indicate that participants felt reassured by their membership in the group, describing it as a resource that could help ease pressures, or *mental load* associated with being a caregiver of a child or youth with CCNs. The emphasis on informational support rather than emotional support, which was reported to be more predominant in condition-specific Facebook groups, resulted in caregivers reporting the group to be a positive space, rather than a reminder of emotional difficulties beyond their control. Other Facebook groups were frequently described as *triggering* negative emotions, whereas the geographical specificity and inclusive nature of this group was perceived by caregivers to be more conducive to the exchange of informational and navigational support.

Most participants in the web-based survey were women and aged 25 to 44 years, which was consistent with previous investigations, which found that women are more likely to engage in P2P support on social media for health-related concerns [53]. Although there was a wide variety of ages and conditions experienced by caregivers, participants felt that the inclusive nature of the group contributed to feeling as if it is a *safe space* for the exchange of P2P support. Findings related to the reported social media use by survey participants, including membership in other Facebook support groups and use of the caregiver support group, have been reported elsewhere [12].

Participants in this study described a lack of control associated with being a caregiver of a child or youth with a CCN. This lack of control was described as a particularly important motivation for seeking Facebook-based P2P support. These findings support previous investigations suggesting an association between participation in P2P support groups and knowledge of health-related resources among caregivers of children with disabilities [54]. The availability and accessibility of the Facebook group was also identified as a reason why participants used the group; many participants described feeling overwhelmed in their role as a caregiver, with very less time.

As the group was closed to caregivers who reside in New Brunswick, there was exclusivity regarding membership that led to some participants valuing a sense of shared cultural norms. Using Facebook support groups to find like-minded people who share similar health practices has been previously observed. For example, Zhang et al [55] noted that members of a Chinese depression support group began using the group to connect with others who shared Chinese health beliefs and practices, which differ from traditional medical practices. The geographical specificity of the group was identified by many participants in this study as a motivating reason for joining, as it offered a notably different experience than condition-specific support groups on the platform with international members.

Approximately all the participants in this study reported difficulties in navigating services and resources related to their child’s care owing to lack of knowledge of relevant services and programs; this was described as a reason for joining the Facebook group. Some interview participants disclosed that they had directly asked for informational support in the group, which, in turn, increased their knowledge of programs or other resources. Others learned about locally available support by passively reading comments or posts by other members. Considerable number of studies has demonstrated an association between offline support groups and increased knowledge among caregivers [56]. The impact of web-based groups is less clear; however, a recent systematic review of the impact of web-based P2P support for caregivers of stroke survivors [57] supports the finding that participation is associated with increased caregiver knowledge.

Despite the short time since the group’s inception, most participants reported feeling a sense of community belonging within the group. The immediate sense of community belonging reported by some members was attributed to the group’s exclusivity, specifically to caregivers of children and youth with CCNs in New Brunswick, despite the diversity in reported ages and conditions. Some participants did not feel a sense of community belonging with the group cited, in part, owing to the short time since the group’s creation. One of these individuals was caring for an older youth and did not identify with other members, most of whom were raising children aged <12 years. This finding corroborates previous observations that a sense of community in web-based groups is facilitated by more homogenous membership [58].

The finding that social belonging was facilitated by group membership may have been owing to the exclusivity of the group. Caregivers reported feeling a sense of solidarity with other members, knowing that they faced similar challenges. The use of web-based groups for coping resources have been attributed to a lower risk of threat to one’s personal social ties compared with the mobilization of offline resources [59]. In other words, although participants in the Facebook group shared many of the same characteristics, such as geographic location, engagement with the group for social support could be obtained even without social interaction (eg, passive interaction). Moreover, the closed (ie, private) nature of the group may have resulted in greater relational intimacy between members, which led to a shared sense of community than if the group had been public [60]. However, this perceived relational intimacy may pose a risk to web-based communities of this nature, whereby reduced nonverbal cues, facilitated by the computer-mediated environment, may result in misplaced credibility or *hyperpersonal* interaction [61]. Specifically, the social information processing theory posits that individuals enter into a loop of intense interpersonal interactions that can lead to the perception that others may be more trustworthy or credible than in actual fact [62]. However, more studies are needed to better understand the effects of hyperpersonal interactions on perception of support providers [63].

Social comparison theory can be used to contextualize some of the findings of this study. Social comparison theory suggests that individuals compare their situations with those of similar others to make assessments about their own health and well-being [64]. Although the evaluation of this theory is limited in the study of web-based support groups [65], it may be applicable to understanding why caregivers may have experienced perceived benefits from participation in this study. Many caregivers reported the perceived benefits of participation, specifically on their knowledge of services and resources and sense of community. Social comparison theory suggests that individuals make lateral, upward, and downward comparisons with others within their social network. Lateral comparisons with similar others may have led to a sense of normalization and comradery between caregivers, thus affecting the perceived sense of community. Upward comparisons occur when individuals compare themselves with others who appear to have more experience or better coping skills; this can lead to inspiration to improve one’s situation and learn from their experiences. In contrast, upward comparisons can result in feelings of frustration. Downward comparisons occur when individuals compare themselves with others who appear to be struggling, which can result in an altruistic desire to share one’s knowledge and experiences. These social comparisons may explain why caregivers perceived benefits as a function of participation in the Facebook group.

### Limitations

Limitations of this study include the small sample size of caregivers of children and youth with CCNs who participated in the Facebook group, particularly in the survey and interviews. The survey and interviews may have oversampled caregivers who are more involved in the care of their child or children. Items in the survey were not validated, and we did not evaluate the reliability of the questions owing to limited time and resources. Moreover, findings from the survey may have been affected by the response rate of 36.1% (39/108). None of the participants in this study identified as male, which would affect the generalizability of the present findings to male caregivers. Participants who participated in the survey and interviews were not independent samples; there was overlap between these 2 subsamples from the Facebook group participants. More specifically, 12 (86%) of the 14 participants who completed the survey also participated in the interview to elaborate on their experiences. It is possible that the explicit emphasis on research within the group (eg, requiring consent to join the group) may have influenced the sample of individuals who joined the group and their subsequent experiences. Individuals who joined the Facebook group were required to undergo screening to ensure that they identified as a caregiver of a child or youth with CCNs and resided in New Brunswick; however, this information was self-reported and could not be verified. Therefore, it is possible that some of the members in the group did not fit the target population of the study. However, all participants in the survey and interviews reported information on their role as caregivers of child or children or youth with CCNs. Finally, the study intervention and investigation were conducted during the COVID-19 pandemic, which has been identified to particularly affect caregivers of individuals with CCNs [5]. It is unclear to what extent the pandemic may have affected the behaviors of caregivers in this study and whether these individuals would have used the group to the same extent. Therefore, the pandemic may have affected the generalizability of these findings.

### Future Studies

This study demonstrated that participation in a closed Facebook group can positively impact the sense of social belonging in a caregiving population that often experiences isolation and exclusion [58]. Moreover, some participants in this study reported learning about health-related and social services and resources that directly affected the care of their child. These findings suggest that Facebook groups, which are low-cost and relatively accessible, can be leveraged to fill the gaps in the support needs of patients and caregivers. However, more studies are needed to systematically determine both positive and negative impact of participation in these groups on these populations. A novel component of this study was the use of a patient navigator as a moderator; although a crowdsourcing effect was observed in this study between caregivers of children and youth with CCNs, the presence of a patient navigator likely may have provided additional information about relevant services or resources or influenced the nature of discussions within the group. Future studies could consider the role and impact of patient navigators and other health professionals on Facebook-based P2P support groups.

Many participants in this study were caregivers of young children with CCNs; future studies are needed to explore how caregivers at different stages of their caregiving journey experience and benefit from web-based P2P support groups. Previous study has suggested that caregivers of young children with CCNs look to caregivers with older children and youth with CCNs to see where their own children may end up [8]; however, findings from this study suggest that some caregivers view this longitudinal perspective as *triggering* and become overwhelmed. More studies are needed to understand this distinction between caregivers of children and youth with CCNs.

Although it is beyond the scope of this project, future studies may explore the impact that participation in web-based P2P support groups may have on offline relationships between caregivers of children and youth with CCNs. More specifically, future studies may consider that knowledge gained from these web-based P2P interactions can influence offline conversations, such as with care providers; this may provide further context into why caregivers use web-based support groups [66].

The findings that participation in the Facebook group was identified by some participants as positively affecting their sense of social belonging was significant, particularly given the short time between the group’s inception and evaluation. Caregivers of children and youth with CCNs often report a sense of isolation and exclusion owing, in part, to significant caregiver burden and disease stigma [65,67]. Combined with high levels of stress and physical exhaustion, this population is at risk of mental health conditions such as anxiety and depression, thus posing an additional risk to the care of their vulnerable child [68,69]. This has become particularly salient during the COVID-19 pandemic, as a result of social distancing measures and fear associated with caring for an immunocompromised child [7]. Improving the sense of social belonging in caregivers of children and youth with CCNs is paramount to ensuring the well-being of both the caregiver and child or youth. This study has important implications for the integration of social media–based support groups into existing organizations and entities that provide health and social support to this population and other patient and caregiver cohorts living with CCNs.

### Conclusions

Social media present an important opportunity to facilitate the exchange of support between patients and caregivers in an accessible and curated environment. Caregivers of children and youth with CCNs engage in web-based P2P support to connect with peers who possess invaluable knowledge gained through lived experiences and exchange support. This study found that caregivers used a geography-specific Facebook group to exchange informational and navigational support in what was perceived as a safe environment. Caregivers of children and youth with CCNs reported social connection with other members within the group, despite a short observation period. This study demonstrated that involvement in web-based support groups can influence perceived knowledge of services and resources and the sense of community belonging, thus helping to meet previously unmet support needs.

## Abbreviations

CCN: complex care need
P2P: peer-to-peer
PFAC: Family and Patient Advisory Council
